# Nitrate deficiency decreased photosynthesis and oxidation-reduction processes, but increased cellular transport, lignin biosynthesis and flavonoid metabolism revealed by RNA-Seq in *Oryza sativa* leaves

**DOI:** 10.1371/journal.pone.0235975

**Published:** 2020-07-10

**Authors:** Cai-Hong Shao, Cai-Fei Qiu, Yin-Fei Qian, Guang-Rong Liu

**Affiliations:** Institute of Soil Fertilizer and Resources Environment, Jiangxi Academy of Agricultural Sciences, Nanchang, China; ICAR-Indian Institute of Agricultural Biotechnology, INDIA

## Abstract

Rice cultivar “Weiyou916” (*Oryza sativa* L. ssp. *Indica*) were cultured with control (10 mM NO_3_^-^) and nitrate deficient solution (0 mM NO_3_^-^) for four weeks. Nitrogen (N) deficiency significantly decreased the content of N and P, dry weight (DW) of the shoots and roots, but increased the ratio of root to shoot in *O*. *sativa*. N deficiency decreased the photosynthesis rate and the maximum quantum yield of primary photochemistry (F_v_/F_m_), however, increased the intercellular CO_2_ concentration and primary fluorescence (F_o_). N deficiency significantly increased the production of H_2_O_2_ and membrane lipid peroxidation revealed as increased MDA content in *O*. *sativa* leaves. N deficiency significantly increased the contents of starch, sucrose, fructose, and malate, but did not change that of glucose and total soluble protein in *O*. *sativa* leaves. The accumulated carbohydrates and H_2_O_2_ might further accelerate biosynthesis of lignin in *O*. *sativa* leaves under N limitation. A total of 1635 genes showed differential expression in response to N deficiency revealed by Illumina sequencing. Gene Ontology (GO) analysis showed that 195 DEGs were found to highly enrich in nine GO terms. Most of DEGs involved in photosynthesis, biosynthesis of ethylene and gibberellins were downregulated, whereas most of DEGs involved in cellular transport, lignin biosynthesis and flavonoid metabolism were upregulated by N deficiency in *O*. *sativa* leaves. Results of real-time quantitative PCR (RT-qPCR) further verified the RNA-Seq data. For the first time, DEGs involved oxygen-evolving complex, phosphorus response and lignin biosynthesis were identified in rice leaves. Our RNA-Seq data provided a global view of transcriptomic profile of principal processes implicated in the adaptation of N deficiency in *O*. *sativa* and shed light on the candidate direction in rice breeding for green and sustainable agriculture.

## Introduction

Nitrogen (N) is one of essential macronutrients required for plant growth and development. It is a constituent of amino acids, nucleic acids, vitamins, chlorophyll, alkaloids, some plant hormones and secondary compounds in plants. N is the most abundant mineral element in plants, which constitutes about 2% - 4% of plant dry weight (DW) [1]. Although some organic form of N, such as urea, amino acids and amides could also be absorbed by plant, ammonium and nitrate are the two predominant forms of N acquired by plant roots from the soil solution. Since these nutrients are scarce in natural soils, N deficiency can be occurred when the total of N absorbed by plant roots from the regime explored by roots less than the N demanded for the growth of plants [2]. Without artificial N fertilizer input, N deficiency is one of most important constraining factors that limited the production and quality of agricultural products.

Absorbed N can be transported through xylem to the leaf canopy as nitrate ions or in its reduced forms, such as amino acids or amides. As N can be re-distributed and transported via phloem in plants, the visible symptoms of N deficiency are the developed chlorosis in older leaves and the significantly lower biomass production [3]. N deficient plants have a short and spindly appearance. In rice, N deficiency leads to poor tillering and small leaf area [1]. In a case of long-term N deficiency, plant leaves turn brown and die, and eventually the production and quality of agricultural products will be reduced. According to the available literatures, N deficiency induced leaf chlorosis and reduction of plant growth have been reported in many plants including rice [4, 5], sweet-potato [6], maize [7], sunflower [8], tobacco [9], rapeseed [10], *Arabidopsis* [11], wheat [12]). Lin et al. reported that N deficiency reduced shoot growth, the contents of nitrate, chlorophyll, protein and ascorbate, and the activities of ascorbate peroxidase, glutathione reductase and catalase, whereas increased the contents of abscisic acid and H_2_O_2_ in leaves of rice seedlings [4]. N deficiency also decreased the CO_2_ assimilation and increased the accumulation of carbohydrates, especially starch, mannitol, sucrose and glucose in olive leaves [13]. In sunflower, N deficiency lead to a higher accumulation of monosaccharides (glucose and fructose) in leaves than control ones, which may be responsible for the faster senescence in the N-deficient plants [8] Sung et al. also reported that N deficiency increased the contents of glucose, fructose and sucrose, and greatly affected the level of shikimate in tomato leaves [14].

As the improvement of molecular and biochemistry techniques, two kinds of N transporter were identified being responsible for the absorption and transportation of N in plants since past decade. Ammonium is transported by transporters of AMT superfamily, all of which have high-affinity ammonium transport activities, except *At*NPF6.3 in *Arabidopsis* and *Os*NPF6.5 in rice, which have dual-affinity transport activity [2, 15, 16]. Nitrate transport is mediated by two families of transporters, named Nitrate Transporter 1/Peptide Transporter Family (NPF or NRT1) and NRT2, members of the former one identified so far have low-affinity transport activity and members of the later one have high-affinity transport activity [17, 18]. Among these two kinds of transporters, Hsu et al. [19] reported that NRT1.11 and NRT1.12 are involved in xylem-to-phloem transfer for redistributing nitrate into developing leaves, and such nitrate redistribution ensures the optimal plant growth under increasing external nitrate. Shin et al., (2018) reported that the expression levels of NRT2.1 and NRT2.2 continued to increase gradually until 5 days after N starvation, and then reduced to un-induced levels at 7 days after N starvation [5]. Furthermore, Lezhneva et al. revealed that together with NRT2.1, NRT2.2 and NRT2.4, NRT2.5 is crucial factor to better growth of nitrogen-starved adult plants by facilitating the efficient uptake of nitrate and involving in nitrate loading into the phloem [20].

Plant hormones have crucial roles in plant growth, development and response to biotic and abiotic stresses. Sun et al. found that N deficiency led to increased seminal root length and decreased lateral root density via stimulating the production of strigolactones and reducing the transport of indole-3-acetic acid from shoots to roots [21]. Garnica et al. reported that the presence of nitrate was associated with clear increases in the active forms of cytokinins, enhanced IAA and lower ABA concentration, independently of the dose applied, demonstrating the possible signal effect of nitrate ion in its beneficial effect on the growth of wheat [22]. Expression of *1-aminocyclopropane-1-carboxylic acid synthase 2* (*OsACS2*, *LOC_Os04g48850*) was modulated in response to N-supplementation in rice, implying that it is important candidate for studying how crop productivity can fluctuate according to nitrogen supply [23]. By using cDNA-amplified fragment length polymorphism, Trevisan et al., found that a new complex signaling framework in which brassinosteroids (BRI1), the module MKK2–MAPK6 and the fine regulation of nitric oxide homeostasis may play key functions in maize responses to nitrate [24].

Rice is one of the most important dairy staple foods worldwide. So far, the nitrogen-use efficiency is relatively low in the fields, especially in eastern Asia. The loss of N in the fields may be caused by the ammonia volatilization, leaching, runoff and denitrification in the case of N. The lost N could severely pollute environment, especially water system [25]. Thus, given the importance of N for plant growth and the environmental cost of intense fertilization, an understanding of the molecular mechanisms underlying the plant adaptation to N deficiency is a primary goal for green and sustainable agriculture. *Oryza sativa* L. ssp. *indica* occupies nearly two-thirds of rice planting acreage in Asia, which frequently went through dry-wet alternate conditions during growing period. Studying the absorption of nitrate and the tolerance of nitrate deficiency in rice is of significant values. RNA-Seq is a powerful tool, which has been used to shed light on the molecular mechanisms underlying plant adaptations to mineral nutrient deficiency. Here, we investigated the physiological and transcriptomic responses to N deficiency in rice seedlings by using biochemical and Illumina sequencing techniques.

## Materials and methods

### Plant culture and N treatment

Uniform seeds of rice (*O*. *sativa* L. ssp. *indica*) cultivar “Weiyou916” were sown in plastic tray containing semidry paddy soil. After germination, robust and uniform seedlings with three leaves and one sprout were transplanted to hydroponic barrel containing adequate Hoagland full-strength nutrient solution. The full strength nutrient solution contained the following macronutrients (in mM): KNO_3_, 5; Ca(NO_3_)_2_, 5; KH_2_PO_4_, 2; MgSO_4_, 2; micronutrients (in μM): H_3_BO_3_, 5; MnCl_2_, 6; ZnSO_4_, 1; CuSO_4_, 0.5; (NH_4_)_6_Mo_7_O_24_, 0.065; and FeSO_4_-EDTA, 20. In N deficient nutrient solution, 5 mM KNO_3_ and 5 mM Ca(NO_3_)_2_ were replaced by 5 mM KCl and 5mM CaCl_2_, respectively. There were three seedlings per tray and grown under natural light condition in Jiangxi Academy of Agricultural Sciences, Jianxi province, China. Two weeks later, seedlings with five leaves were transplanted to new culture hydroponic barrels with sufficient (control) or without nitrogen (N deficiency). Each treatment has ten replicates (barrels). The nutrient solution was refreshed every three days. Four weeks later, top third and forth leaves were collected at noon on a sunny day. All the samples were wrapped in aluminum foil, immersed in liquid nitrogen and stored at −80°C until extraction.

### Measurement of CO_2_ assimilation, chlorophyll a fluorescence, dry weight (DW) and N content

The rate of CO_2_ assimilation was measured by using CIRAS-2 portable photosynthesis system (PP systems, Amesbury, USA). CO_2_ was supplied by a CO_2_ cylinder with a controlled concentration of 360 μmol mol^-1^ and light intensity was set to 1000 μmol m^-2^ s^-1^. CO_2_ assimilation was measured between 9:30 am and 11:45 am on a clear day with environmental temperature and vapor pressure deficit being 29 ± 0.5°C and 1.39 ± 0.1 kPa, respectively. Chlorophyll a fluorescence of dark-adapted leaves was measured by using Handy PEA portable fluorescence analyzer. At the end of experiment, seedlings were harvested and separated into roots and shoots. Plant samples were oven dried to a constant weight at 80°C and then weighed on an electronic balance. For the measurement of N content, grounded leaf and root samples were digested with concentrated H_2_SO_4_-H_2_O_2_ and then tissue N contents were analyzed by using Foss Kjeltec 8200 nitrogen determiner (Hilleroed, Denmark).

### Measurement of soluble carbohydrates, malondialdehyde (MDA) and lignin content

Soluble carbohydrates were extracted with the 80% (v/v) ethanol solution. The residue was used to starch measurement. The extraction was vacuum-dried and the pellet was re-dissolved with three milliliters ultrapure water. The obtained solution was used to the measurement of glucose, fructose and sucrose by using the method described by Yang et al. [26]. MDA (represented as TBARS) were extracted was extracted and measured according to the methods described by Hodges et al. [27]. Leaf samples from different N treatments were homogenized with 95% (v/v) ethanol solution. The extraction was discarded after centrifugation at 2000 *g* for 3 min and the pellet was washed three times with 95% (v/v) ethanol and two times with ethanol-hexane (1:2, *v*/*v*). The washed pellet was oven-dried at 50°C. The lignin content was determined according to the method described by Morrison [28]. There were four replicates for soluble carbohydrates, MDA and lignin contents.

### Total RNA extraction and construction of cDNA library for high throughput sequencing

Samples from four different plants were harvested and pooled into one biological replicates that used to RNA extraction. Total RNA were extracted from leaf samples by using TRIzol reagent (Invitrogen, Carlsbad, CA). Integrity and purity of total RNA were examined by 1% agarose electrophoresis. The concentration of total RNA was measured by Nanodrop ND2000 (Thermo Scientific, Waltham, USA). The RNA samples with the ratio of A260 (absorbance at 260 nm) to A280 between 1.85 and 2.10 were qualified to further analysis. Message RNA (mRNA) was enriched from 4 μg total RNA by using magnetic beads linked with oligo dT. The obtained mRNA was fragmented into short fragments and used to the first-strand cDNA synthesis and second strand cDNA synthesis. The resulting double-strands cDNA was purified by AMPure XP beads. Purified double-strands cDNA were then used to end repair, 3' end adenosine tailing, sequencing adaptor connection, fragment selection and finally PCR amplification to generate sequencing library. The library quality was evaluated by using the Agilent Bioanalyzer 2100 system (Agilent, Santa Clara, CA 95051, USA) and ABI Step-One-Plus real-time PCR system. After cluster generation, the cDNA clusters were sequenced on the Illumina HiSeq 2000 platform (Illumina Inc., San Diego, CA, USA) using the paired-end technology and sequencing by synthesis (SBS) method. Illumina GAPipeline (Version 1.3) was used to perform the original image process to sequences, base-calling and alignment to reference sequences, in which 90 bp/90 bp paired-end (PE90) reads were generated. The RNA-Seq data was submitted to NCBI database (https://www.ncbi.nlm.nih.gov/sra/) with SRA accession number PRJNA558244.

### Identification of differentially expression genes (DEGs) and bioinformatic analysis

Raw reads were processed by SOAPnuke software to remove adapters, reads with more than 5% uncertain base and low quality reads (the proportion of Q20 more than 20%). After get rid of the reads mapping to ribosomal RNA, the remaining reads were mapped to *Oryza sativa* genome published on NCBI (https://www.ncbi.nlm.nih.gov/assembly/GCF_001433935.1) by Bowtie2 software [29]. The gene expression level was calculated by FPKM (Fragments Per Kilobase of transcript per Million mapped reads) method [30]. Genes with a FDR value < 0.001 and the absolute value of log_2_|(fold change)| >1 identified by DESeq software were considered DEGs [31]. Gene ontology and KEGG annotation of DEGs was performed using the method described by Young et al. [32] and Kanehisa et al. [33].

### Validation of RNA-Seq data by RT-qPCR

Total RNA was extracted from different leaf samples by using TRIzol reagent (Invitrogen, Carlsbad, CA) and digested by DNase I (Promega, Madison, USA). Quality and quantity of total RNA were monitored by electrophoresis in 1% agarose gel and a Nanodrop 2000 spectrophotometer at the wavelength of 260 nm, respectively. Only high quality RNA was submitted to the subsequent analysis. First strand cDNA was synthesized by using the RevertAid^TM^ First-Strand cDNA Synthesis Kit (Thermo Scientific, Waltham, USA) following the manufacturer’s instructions. Gene special primer pairs were designed according to the sequence of candidate DEGs by using Primer Premier (Version 6.0) and listed in S1 Table. RT-qPCR was performed by using GoTaq^®^ qPCR Master Mix (Promega, Madison, USA). The 20 μL reaction mixture contained 10 μL GoTaq^®^ qPCR master mix, 2 μL cDNA template, 0.2 μmol L^-1^ gene special primer pairs. RT-qPCR was performed by using a CFX96 Touch^TM^ Real-Time PCR System (Bio-Rad, Hercules, CA, USA). The cycling conditions were 60 s at 94°C, followed by 40 cycles of 94°C for 10 s, 68°C for 30 s. Gene expression levels were calculated by using ddCT method. In order to normalize the difference among the samples and replicates, the ubiquitin-60S ribosomal protein gene (accession number: LOC4347896) was used as internal standard and the control sample (N replete) was used as reference, of which the gene expression levels were set to 1.

### Experimental design and statistic analysis

There were ten replicates for each N treatments and each replicate contained three seedlings. There were five replicates for the measurement of CO_2_ assimilation and chlorophyll a fluorescence, eight replicates for plant dry weight, four replicates for N content, soluble carbohydrates, MDA and lignin contents, respectively. Data were represented as means ± SD (n = 4–8). Means were separated by the student's *t*-test at *p* < 0.05.

## Results

### Effects of N deficiency on the dry-weight (DW), ratio of root to shoot and N content of *O. sativa*

**Fig 1:**
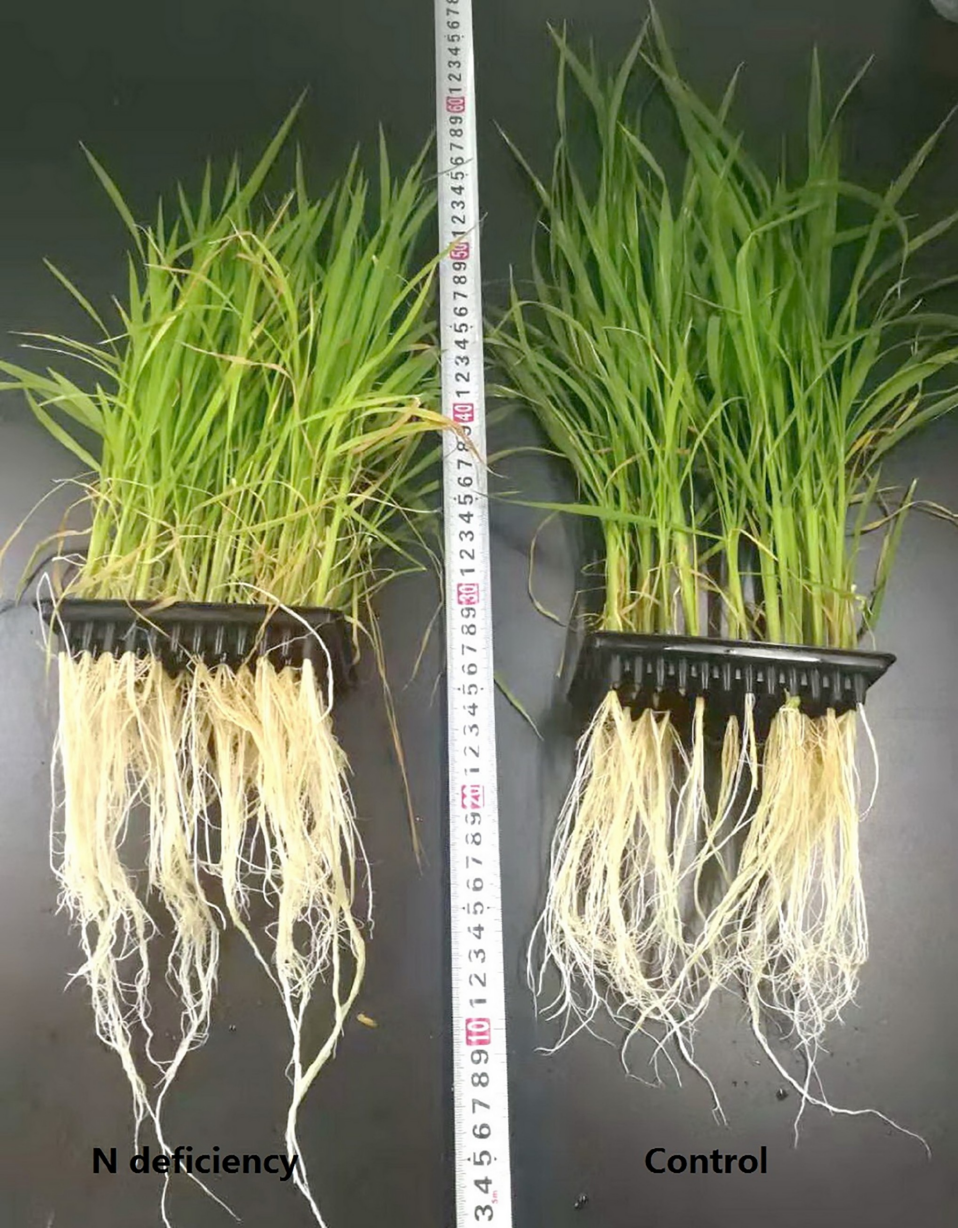
Appearance of *O*. *sativa* seedlings under N deficiency.

**Fig 2:**
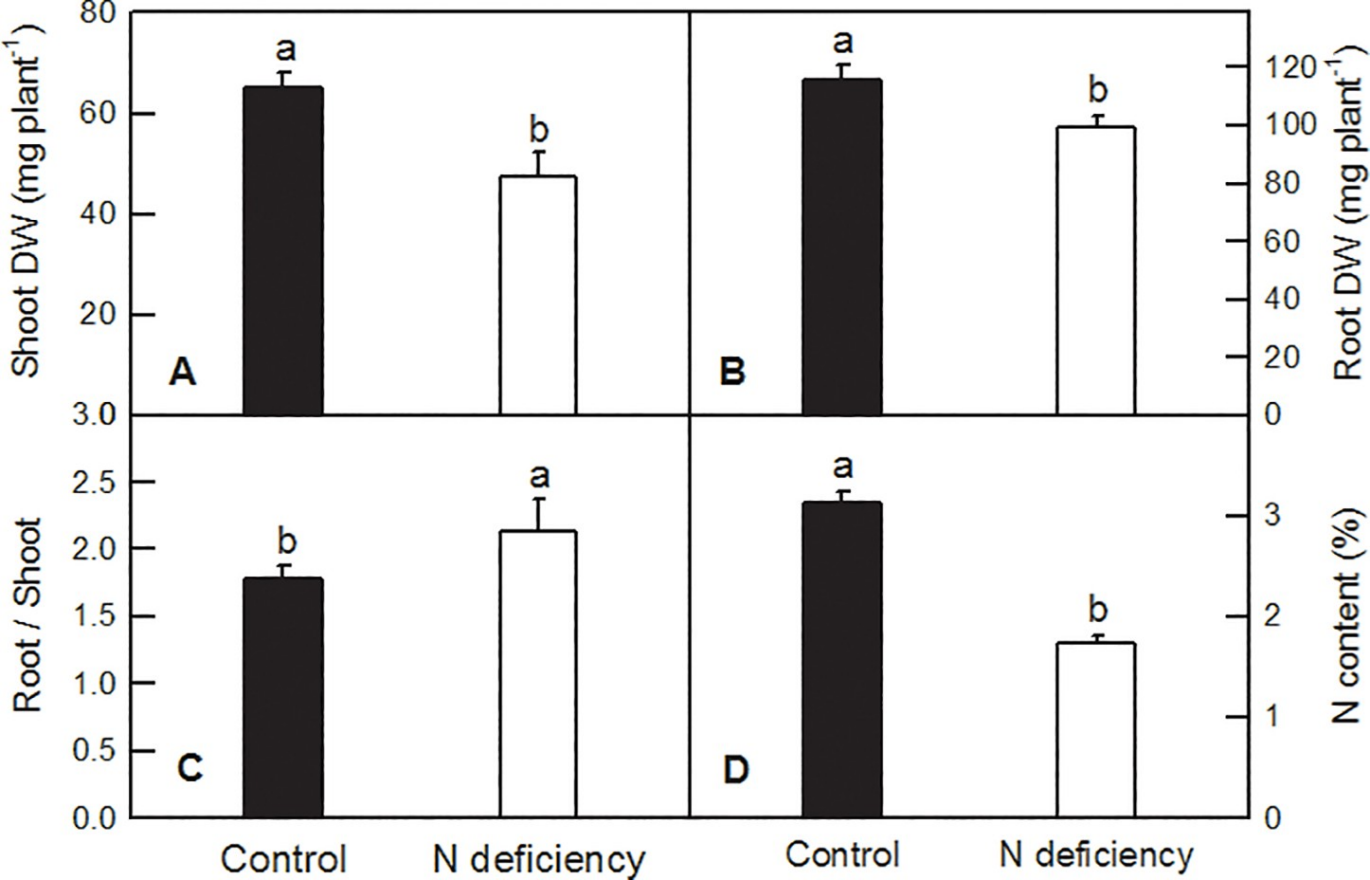
Effects of N deficiency on DW (A and B), root/shoot (C) and N content (D) in *O*. *sativa*. Bars represent means ±SD (*n* = 4 for N content or 8 for plant DW). Difference among the treatments was analyzed by student’s *t*-test. Different letters above the bar indicate a significant difference at *p* < 0.05.

After four weeks treatment, N deficiency led to chlorosis of lower leaves and lowered the height of rice seedlings, when compared to the control ones (Fig 1). N deficiency significantly decreased the DW of shoot and root in rice seedlings by 27.17% and 13.84%, respectively (Fig 2A and 2B), but increased the ratio of root to shoot by 19.29% (Fig 2C). N deficiency dramatically reduced the N content in rice seedlings by 44.66%, when compared to the control ones (Fig 2D).

### Effects of N deficiency on the photosynthesis parameters and soluble carbohydrate contents in *O. sativa* leaves

**Fig 3:**
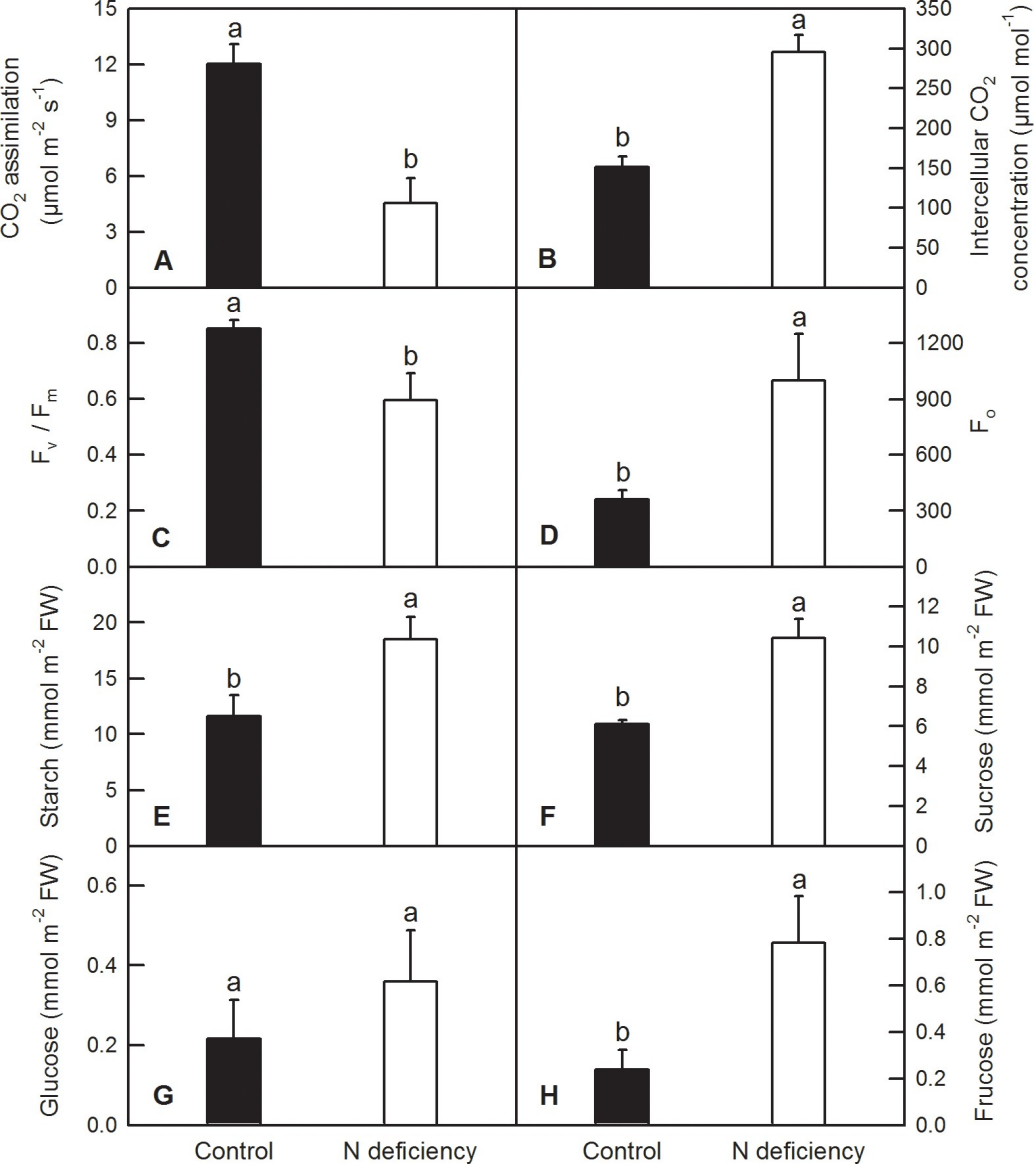
Effects of N deficiency on photosynthesis parameters (A and B), chlorophyll a fluorescence (C and D) and soluble carbohydrate contents (D–H) in *O*. *sativa* leaves. Bars represent means ±SD (*n* = 5 for photosynthesis parameters and chlorophyll a fluorescence, or 4 for soluble carbohydrate contents). Difference among the treatments was analyzed by student’s *t*-test. Different letters above the bar indicate a significant difference at *p* < 0.05.

Gas exchange and chlorophyll a fluorescence measurement showed that N deficiency decreased the CO_2_ assimilation rate by 62.33% (Fig 3A) and the maximum quantum yield of primary photochemistry after dark-adaptive (F_v_/F_m_) by 30.07% (Fig 3C). However, N deficiency increased the intercellular CO_2_ concentration by 95.04% (Fig 3B) and the value of primary fluorescence (F_o_) by 176.71% (Fig 3D). Non-structural carbohydrates, such as starch, glucose, fructose and sucrose, are the main products of photosynthesis. Soluble carbohydrate measurement showed that N deficiency significantly increased the contents of starch, sucrose and fructose by 59.98%, 70.84 and 230.36% respectively, but did not change that of glucose in *O*. *sativa* leaves (Fig 3E–3H).

### Effects of N deficiency on the H_2_O_2_ production, MDA, soluble protein and lignin contents in *O. sativa* leaves

**Fig 4:**
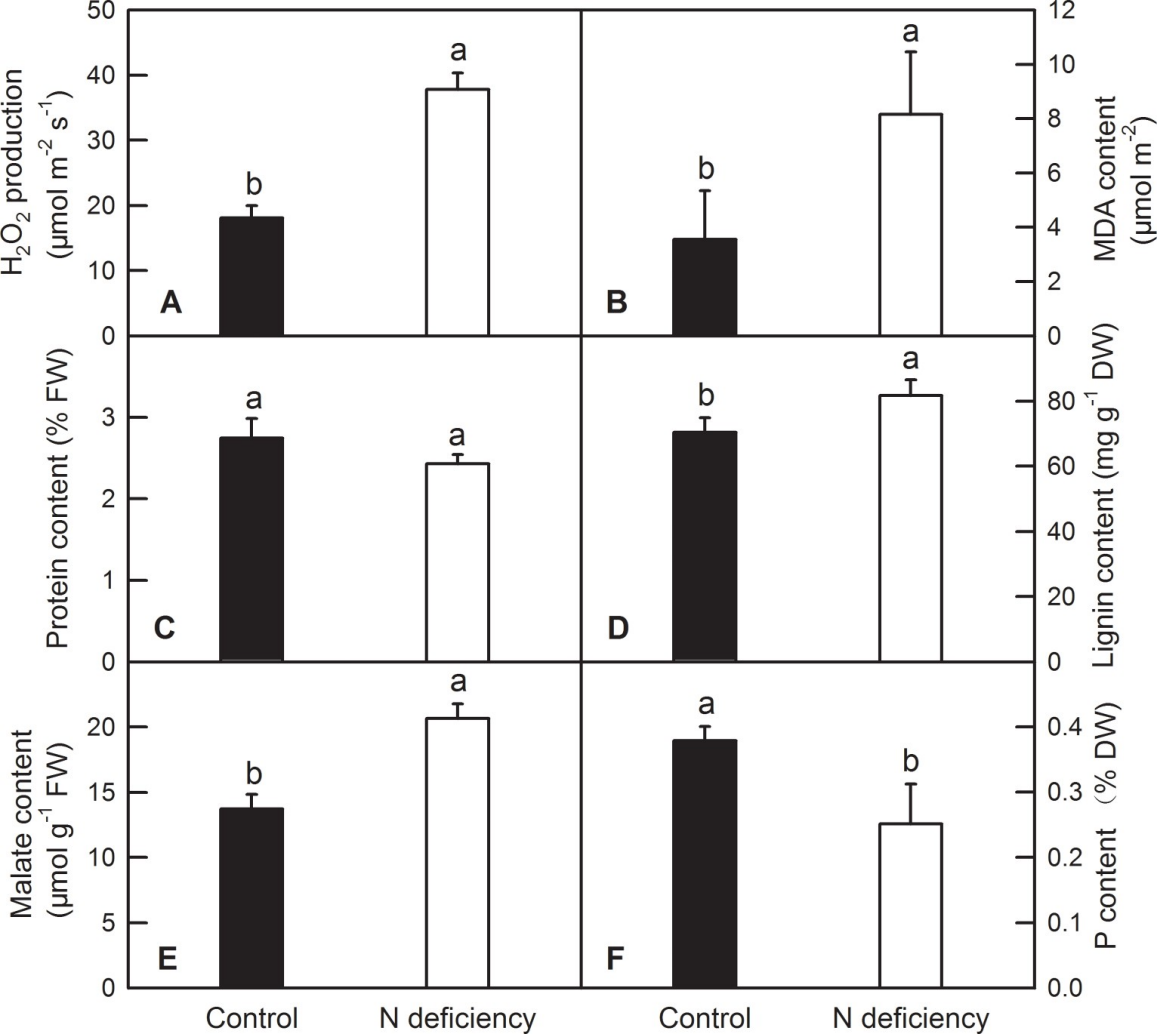
Effects of N deficiency on H_2_O_2_ production, MDA, soluble protein and lignin contents in *O*. *sativa* leaves. Bars represent means ±SD (*n* = 4). Difference among the treatments was analyzed by student’s *t*-test. Different letters above the bar indicate a significant difference at *p* < 0.05.

N deficiency significantly increased the production of H_2_O_2_, MDA content revealed by TBA reactive substance (TBARS) content, lignin and malate content in *O*. *sativa* leaves by 129.69%, 16.05%, 50.59%, respectively (Fig 4A–4E). Decreased photosynthesis led to excess photon absorbed by leaf, which inevitably induce the production of H_2_O_2_ and eventually increase membrane peroxidation as shown in increased MDA content. N deficiency did not change the total soluble protein content in *O*. *sativa* leaves (Fig 4C). N deficiency significantly decreased the content of P in *O*. *sativa* leaves by 33.54% (Fig 4F).

### RNA-Seq, de novo assembly, gene annotation and differentially expressed genes (DEGs) identification in *O. sativa* leaves

After sequencing libraries were constructed and sequenced, raw reads were processed by SOAPnuke software to remove adapters, reads with more than 5% uncertain base and low quality reads were removed. There were 51,606,660 and 51,151,230 total clean read obtained in the control and N deficiency library, respectively (S2 Table). After get rid of the reads mapping to ribosomal RNA, the remaining reads were mapped to *Oryza sativa* genome published on NCBI using Bowtie2 software. Finally, control and N deficiency library gained 41,443 and 42,738 distinct singletons, respectively. The gene expression level was calculated by FPKM (Fragments Per Kilobase of transcript per Million mapped reads) method. According to the criteria of |log_2_(fold change)| > 1 and FDR < 0.001, a total of 1635 DEGs were identified in response to N deficiency in *O*. *sativa* leaves. Of which, 999 DEGs and 636 DEGs were downregulated and upregulated by N deficiency in *O*. *sativa* leaves, respectively (S3 Table). Using blast2go software, these DEGs were annotated to GO term and 195 DEGs were found to highly enrich in nine GO terms (S4 Table). The enriched GO term were list as follows: photosynthesis (GO:0015979), photosynthesis, light harvesting (GO:0009765, GO:0009768), transmembrane transport (GO:0055085), metal ion transport (GO:0030001), phosphate ion transport (GO:0006817), protein-chromophore linkage (GO:0018298), cellular response to phosphate starvation (GO:0016036), oxidation-reduction process (GO:0055114), flavonoid biosynthetic process (GO:0009813). Hereafter, we mainly focused on the enriched DEGs and the biological processes (GO terms) of which involved in.

### RT-qPCR analysis of selected DEGs in *O. sativa* leaves under N deficiency

In order to verify the RNA-Seq data, we designed the gene special primer pairs and performed RT-qPCR of 16 selected DEGs response to N deficiency in *O*. *sativa* confirmed by RNA-Seq and bioinformatic analysis. These 16 selected DEGs will be discussed in the following discussion part. RT-qPCR results showed that the expression patterns of all the 16 selected DEGs were highly consistent with the results obtained by RNA-Seq, which means that RNA-Seq and subsequent bioinformatic analysis were reliable and robust technique to identify and quantify the differentially regulated genes in response to N limitation in *O*. *sativa* leaves.

## Discussion

### N deficiency decreased light harvesting and photosynthesis rate, but increased soluble carbohydrates accumulation in *O. sativa* leaves (GO:0015979, GO:0009765, GO:0009768)

**Fig 5:**
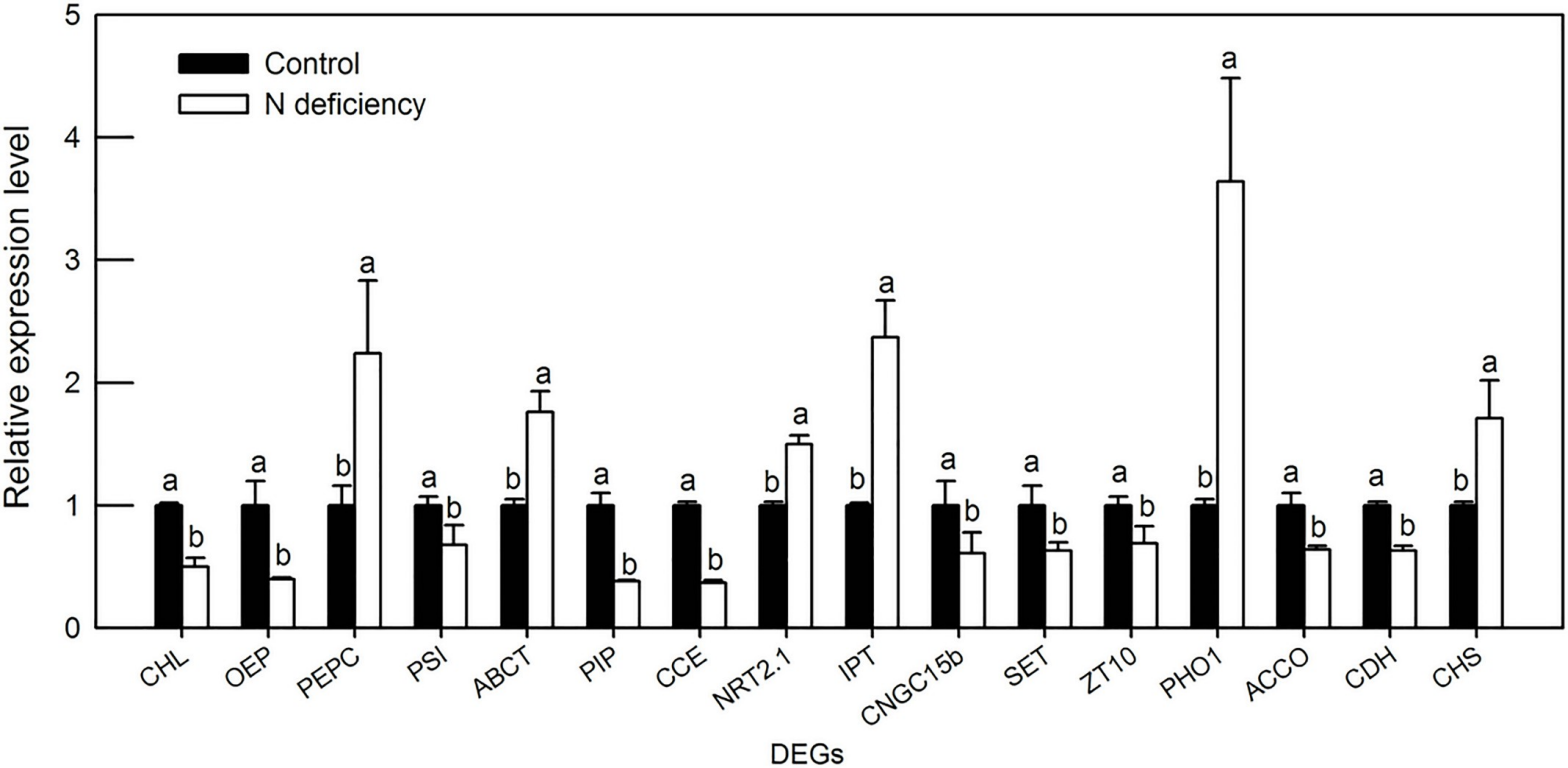
Verification of RNA-Seq data by RT-qPCR analysis of selected DEGs in *O*. *sativa* leaves under N deficiency. *CHL*: Chlorophyll a-b binding protein 1 (0.986); *OEP*: oxygen-evolving enhancer protein 2 (0.938); *PEPC*: phosphoenolpyruvate carboxylase 2 (0.868); *PSI*: photosystem I chlorophyll a/b-binding protein 2 (0.86); *ABCT*: ABC transporter G family member 35 (0.965); *PIP*: aquaporin PIP 1-3-like (0.983); *CCE*: cation/calcium exchanger 1 (0.999); *NRT2*.*1*: high-affinity nitrate transporter 2.1 (0.985); *IPT*: probable inorganic phosphate transporter 1–3 (0.97); *CNGC15b*: protein CNGC15b (0.97); *SET*: silicon efflux transporter LSI2-like (0.89); *ZT10*: zinc transporter 10 (0.878); *PHO1*: phosphate transporter PHO1-3-like (0.939); *ACCO*: 1-aminocyclopropane-1-carboxylate oxidase 1-like (0.95); *CDH*: cytokinin dehydrogenase 4-like (0.99); *CHS*: chalcone synthase 1-like (0.89). Bars represent means ± SD (n = 3). Difference among the treatments was analyzed by student’s *t*-test. Different letters indicate a significant difference at *p* < 0.05. The number in the parenthesis behind protein name is correlation coefficiency between RT-qPCR and transcriptome data of each gene.

As N is a reusable and transferable nutrient in plant phloem, the chlorosis symptom appears firstly in mature leaves or the leaves near the growing point (Fig 1). Leaf chlorosis induced by N deficiency has been reported in many plants including sweet-potato [5], maize [7, 34], olive [13], tobacco [9], rapeseed [10], *Arabidopsis* [11], wheat [12], rice [23, 35–37] and so on. Sinha et al. reported that the photosynthetic pigments showed a drastic reduction in rice cultivar N22 under low N, while cultivar IR64 was more resilient [38]. However, knowledge about molecular mechanism underlying N-deficiency-induced leaf chlorosis is relatively scarce. In the current study, we found that N deficiency downregulated most of DEGs involved in photosynthesis and light harvesting process, with 45 DEGs downregulated and only two DEGs upregulated (S4 and S5 Tables). Of these DEGs, the most remarkable DEGs were chlorophyll a/b binding proteins, photosystem I reaction center subunits, photosystem II reaction center subunits, ribulose bisphosphate carboxylase small chain A and chlorophyll metabolism related magnesium-dependent enzymes, such as magnesium-protoporphyrin IX monomethyl ester [oxidative] cyclase and magnesium-chelatase subunit ChlH (S4 Table; Fig 5). This is consistent with the decreased CO_2_ assimilation, maximum efficiency of light energy conversion (F_v_/F_m_), and increased intercellular CO_2_ and chlorosis symptom in *O*. *sativa* leaves under N limitation (Figs 1 and 3A–3C). Similarly, Huang et al. (2016) reported that a chlorophyll a-b binding protein 2 (CAB2) encoding gene (*Os01g0720500*) was downregulated by low N [39]. The decreased photosynthesis is probably a direct effect of accumulated soluble carbohydrates and starch due to their metabolite feedback regulation, as revealed by increased contents of starch, sucrose and fructose in *O*. *sativa* leaves (Fig 3E and 3F). However, Hermans et al. suggested that some of effects of N deficiency on plant seem to be related to C:N ratio rather than the soluble carbohydrate status alone [40].

N deficiency also downregulated the expression levels of genes encoding oxygen-evolving enhancer protein 2 and 3, which were located in photosystem II and responsible for H_2_O photolysis and oxygen evolution. The down-regulation of oxygen-evolving enhancer protein genes may decrease photosystem II integrity, which reflected in the increased F_o_ value in N deficient *O*. *sativa* leaves (S4 and S5 Tables; Figs 3D and 5) [41]. To our best knowledge, this is the first time that DEGs involved in oxygen-evolving complex were reported in response to N deficiency in rice. The downregulated oxygen-evolving enhancer protein was also found in wheat under salt treatment, grape under Cu stress and tea plants under low phosphorus status [42–44], which demonstrated that the deleterious effects of adverse condition on the oxygen-evolving complex in photosystem II might be a common phenomena in plants.

Phosphoenolpyruvate carboxylase (PEPC; EC 4.1.1.31), which irreversibly catalyzes the conversion of phosphoenolpyruvate and HCO_3_^-^ to oxaloacetate (OAA) and inorganic phosphate, is a vital enzyme that functions in primary metabolism [45]. PEPC is also supposed to play a crucial role in modulating the balance of C and N metabolism in *Arabidopsis* leaves [45]. In the current study, a gene encoding phosphoenolpyruvate carboxylase 2 was upregulated by N deficiency in *O*. *sativa* leaves (S4 and S5 Tables). In contrast, the expression level of a PEPC fluctuated with duration of N limitation in rice leaves [39]. Accordingly, the malate (synthesized from OAA by cytosolic NAD-dependent malate dehydrogenase) content was also induced by N deficiency in *O*. *sativa* leaves (Fig 4E). Malate and/or citrate content was also increased by N, P, K deficiency in tomato leaves [14], N deficiency in *Chlamydomonas reinhardtii* and *Arabidopsis* [11, 46]. However, some literatures reported that N deficiency decreased the demand of carbon skeleton represented as organic acids due to the decreased synthesis of amino acids [47, 48]. This discrepancy may be arisen from different duration time of N deficiency or different sampling time points as organic acids diurnally changed in plant leaves.

### N deficiency enhanced cellular transport including nitrate and inorganic phosphorus transport (GO:0055085, GO:0030001, GO:0006817, GO:0016036)

N deficiency differentially regulated 71 DEGs involved in cellular transport in *O*. *sativa* leaves, among which, 41 DEGs and 30 DEGs were upregulated and downregulated, respectively (S4 Table). For instance, N deficiency upregulated the expression levels of genes encoding ABC transporter family members, high-affinity nitrate transporter 2.1, transporters PHO1 members, metal-nicotianamine transporters and sugar transport protein MST3-like, whereas downregulated the expression levels of genes encoding aquaporin PIP members, cation/calcium exchanger, protein CNGC15b, silicon efflux transporters, zinc transporter members, heavy mental-associated isoprenylated plant protein members, etc. (S4 and S5 Tables).

ABC transporter superfamily is a large and ubiquitous group of proteins that mediate magnesium-ATP-energized transmembrane transport and/or regulate other transporters [49]. ABC transporter family proteins have frequently shown to be involved in a great many biological processes such as metal transport, pathogen response, secondary compounds transport, lipid deposition, phytate accumulation, organic acids exudation and auxin transport. Therefore, ABC transporter family proteins play an important role in plant growth, nutrition, development, response to abiotic stress, and the interaction with its environment [49, 50]. Here, we reported that most of differentially regulated ABC transporter family proteins were upregulated by N deficiency, which means that as a nutritional signal, N deficiency may accelerate transmembrane transport, mainly efflux of metabolites in older leaves as a strategic response to recycle useful compounds (S4 and S5 Tables; Fig 5). High-affinity nitrate transporter (NRT) was reported to be induced by N limitation and itself mediated lateral root primordial development in plant roots [51]. Except for nitrate absorption of nitrate anion in plant roots, NRTs were also implicated in nitrate re-allocation among leaves [16]. Some transcriptomic analysis found that N limitation upregulated genes involved in N assimilation and metabolism, such as nitrate reductase (*NR*), nitrite reductase (*NiR*), glutamine synthetase (*GS*), glutamate synthase (*GOGAT*), glutamate dehydrogenase (*GDH*), ammonium transporter 1 (*AMT1*) and also *NRT1* and *NRT2* [35, 37]. However, Shin et al. reported that expression levels of NRT2.1 and NRT2.2 continued to increase gradually until 5 days after N starvation, and then reduced to un-induced levels at 7 days after N starvation [5]. Here, we showed that the expression levels of two *NRT2*.*1* genes were significantly induced by N limitation in *O*. *sativa* leaves (S4 and S5 Tables; Fig 5). The up-regulation of NRT2.1 may facilitate more efficient nitrate utilization in shoots of *O*. *sativa* under N limited condition. On the other hand, the induction of these two transporter genes might also indicate the altered interaction between *O*. *sativa* leaves and pathogen as Camanes et al. showed that the *nrt2*.*1* mutant of *Arabidopsis* shows reduced susceptibility to the bacterial pathogen *Pseudomonas syringae* [52]. However, the possible diverse functions of NRTs in higher plant leaves need to be further investigated.

Aquaporins are members of major intrinsic proteins encompassed PIPs (plasma membrane intrinsic proteins), TIPs (tonoplast intrinsic proteins), NIPs (NOD26-like intrinsic proteins), and SIPs (small basic intrinsic proteins), that selectively facilitate water and small neutral molecules across biological membranes [53]. In the current study, N deficiency downregulated the expression levels of five *PIPs* and one *TIP*, and upregulated one *TIP* gene which located in tonoplast (S4 and S5 Tables; Fig 5). Such result means that N deficiency decreased the demand of water as decreased photosynthesis and stomatal conductance (Fig 3A) [54]. Similarly, N deficiency also downregulated the expression levels of genes encoding cation/calcium exchanger, protein CNGC15b, silicon efflux transporters, zinc transporter members, etc. (S4 Table), which might indicate that the influx of calcium and zinc, and the efflux of silicon will be decreased by N deficiency in *O*. *sativa* leaves. Beside N, phosphorus (P) is another essential nutrient that is required for plant normal growth, development and reproduction. It is also a major ingredient of the fertilizers required to maintain high-yield agriculture [55]. Interestingly, N deficiency upregulated the expression levels of genes encoding probable inorganic P transporter 1–5, P transporter PHO1-1-like protein, and ten genes involved in cellular response to phosphate starvation (S4 and S5 Tables; Fig 5). The upregulation of *PHO1* were also found in rice roots and shoots under N limitation [5]. The plant leaf PHO1 mediated the efflux of phosphate out of cells and into the xylem vessel, revealing a crucial role for PHO1 in phosphate redistribution in plant shoots [56]. Our RNA-Seq data demonstrated that N deficiency may accelerate the efflux of phosphate from mature leaves under N limitation as revealed by lower P content in N deficient leaves (Fig 4F), which in turn significantly upregulated the expression levels of genes related to phosphate starvation.

### N deficiency downregulated most of DEGs involved in oxidation-reduction process (GO:0055114)

Among the DEGs involved in oxidation-reduction process, N deficiency downregulated several genes encoding anti-oxidant enzymes, including cytochrome P450s, ferredoxin-1, heat stress transcription factor A-2c-like, peroxidase 50 and probable L-ascorbate peroxidase 8 (S4 and S5 Tables). As we mentioned above, N deficiency decreased photosynthesis rate and light utilization revealed as lower CO_2_ assimilation and F_v_/F_m_ in N deficient *O*. *sativa* leaves (Fig 3A and 3C), the excess light will inevitably trigger higher production of ROS [57]. The down-regulation of genes encoding anti-oxidant enzymes and the possible up-regulation of ROS production could break the balance of ROS dynamic, which was shown in the higher H_2_O_2_ production and MDA content in N deficient leaves than in the control ones of *O*. *sativa* (Fig 4A and 4B). Plant hormone, including ethylene, auxin and cytokinins, involved in the processes of morphogenesis, regulation of growth, plant interaction with environment and in response to soil nutrient imbalances [40]. Here, we found that the expression levels of genes encoding 9-cis-epoxycarotenoid dioxygenase (NCED1), 1-aminocyclopropane-1-carboxylate oxidase 1 and gibberellin 2-bata-dioxygenase 1, which catalyzed the final step of abscisic-acid, ethylene and gibberellins biosynthesis, respectively, were downregulated by N deficiency in *O*. *sativa* leaves (S4 and S5 Tables; Fig 5). This result demonstrated that the biosynthesis of ethylene and gibberellins may slow down in N deficient rice leaves.

Cytokinins are involved in systemic N deficiency (demand) signaling and likely to act as both local and systemic signals coordinating N demand and acquisition [2]. Cytokinin dehydrogenase is a flavoprotein that cleaves off the cytokinin side chain to yield adenine or adenosine and an aldehyde [58]. In the current study, two genes (cytokinin dehydrogenase 4-like and cytokinin dehydrogenase 5-like) encoding cytokinin dehydrogenase, were significantly downregulated by N deficiency (S4 and S5 Tables; Fig 5). The down-regulation of cytokinin dehydrogenase genes might be a strategic response to N deficient condition as a positive correlation between tissue cytokinin content and N availability has been reported in many plant species including rice [2, 59].

Interestingly, we found that several genes, such as laccase-5, laccase-6, laccase-9, probable cinnamyl alcohol dehydrogenase 6 and probable cinnamyl alcohol dehydrogenase 8D, which involved in the biosynthesis of lignin, were significantly upregulated by N deficiency in *O*. *sativa* leaves (S4 and S5 Tables). The up-regulation of genes involved in the biosynthesis of lignin is consistent with the increased lignin contents in N deficient *O sativa* leaves (Fig 4D). The possible mechanism of upregulated lignin biosynthesis might be due to the higher contents of soluble carbohydrates (Fig 3E, 3F and 3H) and H_2_O_2_ production (Fig 4A), which can be converted to lignin via tricarboxylic acid cycle (TCA)/ phenylpropanoids pathway, laccase and/or peroxidase (POD) /H_2_O_2_, respectively. Such phenomenon was also observed in other crops under nutrient disorders, such as tobacco under excess boron [60], citrus under Mg deficiency [61] and *Pinus massoniana* under P deficiency [62], respectively. However, N-deficiency-induced DEGs involved in lignin biosynthesis were rarely reported in previous transcriptomic studies in rice.

### N deficiency increased the DEGs involved in flavonoid biosynthetic process (GO:0009813)

Flavonoids (flavanols, flavonols and anthocyanins), which have antioxidant activity, are the largest class of phenolics. They are resulted from the addition of malonyl CoA to the phenylpropanoid molecule coumaroyl CoA and are widely distributed plant secondary metabolites [63]. Many genes involved in flavonoid biosynthesis are induced under stress conditions and considerable literatures found that there was significantly increase in flavonoid levels in plants suffered biotic and abiotic stresses, such as high pH, N deficiency, wounding, drought, metal toxicity and nutrient deprivation [64–66]. Here, our RNA-Seq data showed that the expression levels of genes such as anthocyanidin reductase, bisdemethoxycurcumin synthase-like, chalcone synthase (*CHS*), chalcone-flavonone isomerase (*CHFI*), flavanone 3-dioxygenase 2-like, etc., which implicated in flavonoids biosynthesis were apparently upregulated by N deficiency in *O*. *sativa* leaves (S4 and S5 Tables; Fig 5). Huang et al. (2016) also reported that the expression level of CHS was induced by low N in rice by microarray hybridization technique [39]. Peng et al. reported that N limitation could channel the phenylpropanoid metabolic flux to the induced anthocyanin synthesis, which facilitated the adaptation of *Arabidopsis* seedlings to N limitation [67]. More recently, Randriamanana et al. and Song et al. found that both low N and low P increased the amount of flavonoids and other phenylpropanoids in European aspen and polar, respectively [68, 69]. Therefore, the up-regulation of genes involved in flavonoids biosynthesis could be beneficial to rice seedlings under N limitation.

## Conclusions

**Fig 6:**
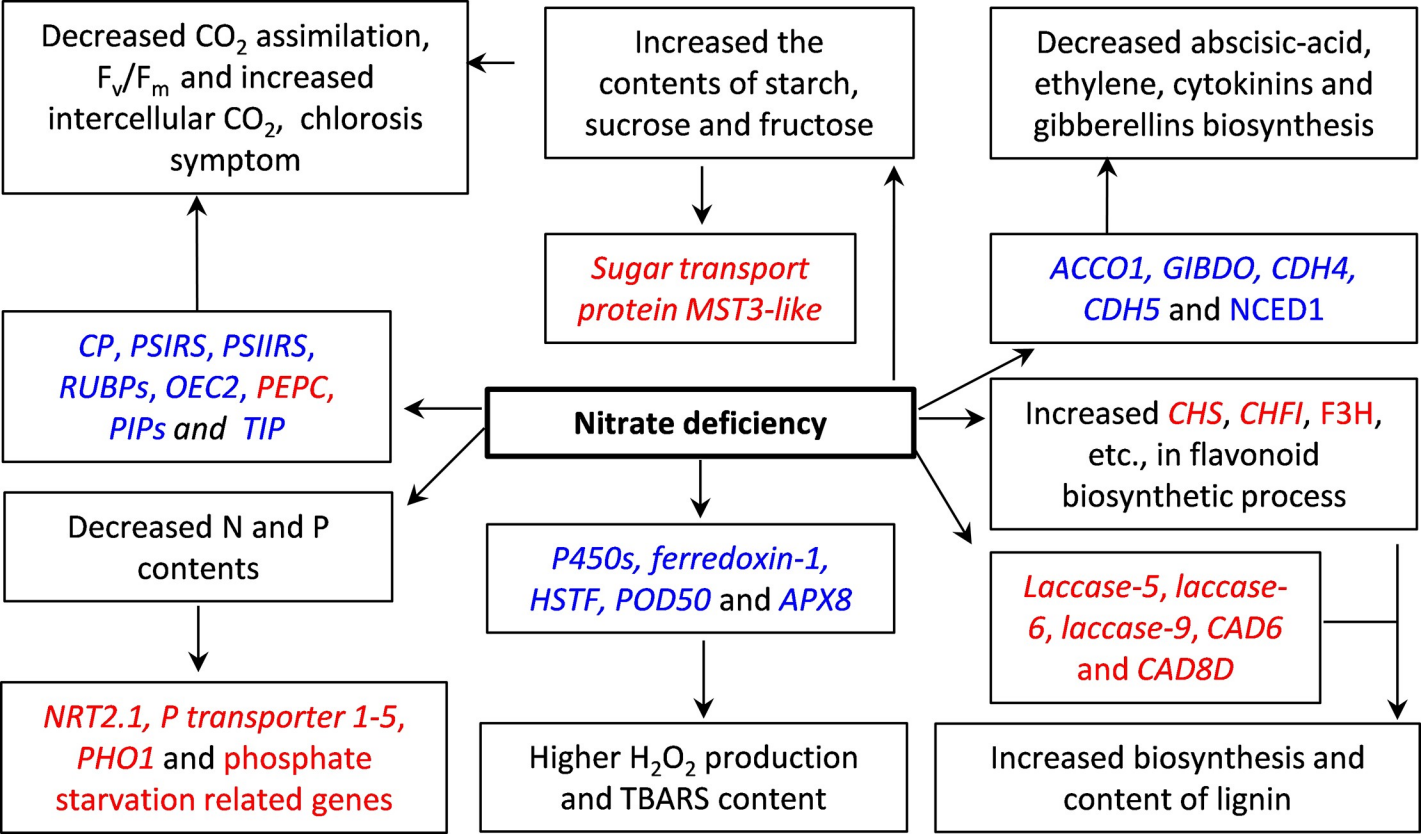
Diagram of effects of N-deficiency on major pathways of *O*. *sativa* leaves. Chlorophyll a/b binding proteins, *CP*; photosystem I reaction center subunits, *PSIRS*; photosystem II reaction center subunits, *PSIIRS*; ribulose bisphosphate carboxylase small chain A, *RUBPs*; oxygen-evolving enhancer protein 2, *OEC2*; Phosphoenolpyruvate carboxylase, *PEPC*; High-affinity nitrate transporter 2.1, *NRT2*.1; cytochrome P450s, *P450s*; heat stress transcription factor A-2c-like, *HSTF*; peroxidase 50, *POD50*; probable L-ascorbate peroxidase 8, *APX8*; 1-aminocyclopropane-1-carboxylate oxidase 1, *ACCO1*; gibberellin 2-bata-dioxygenase 1, *GIBDO*; Cytokinin dehydrogenase 4-like, *CDH4*; cytokinin dehydrogenase 5-like, *CDH5*; 9-cis-epoxycarotenoid dioxygenase, *NCED1*; chalcone synthase, *CHS*; chalcone-flavonone isomerase, *CHFI*; flavanone 3-dioxygenase 2-like, *F3H*; cinnamyl alcohol dehydrogenase 6, *CAD6*; cinnamyl alcohol dehydrogenase 8, *CAD8*.

N deficiency significantly decreased the content of N, DW of the shoots and roots, but increased the ratio of root to shoot in *O*. *sativa*. N deficiency decreased the photosynthesis and F_v_/F_m_, however, increased the intercellular CO_2_ concentration and F_o_. N deficiency significantly increased the production of H_2_O_2_, the contents of MDA, lignin, starch, sucrose and fructose, but did not change that of glucose in *O*. *sativa* leaves. A total of 1635 genes showed differential expression in response to N deficiency revealed by RNA-Seq and the transcriptomic data were further validated by RT-qPCR. Of which, 999 DEGs and 636 DEGs were downregulated and upregulated by N deficiency in *O*. *sativa* leaves, respectively. GO analysis showed that 195 DEGs were found to highly enrich in nine GO terms. Most of DEGs involved in photosynthesis, biosynthesis of ethylene and gibberellins were downregulated, whereas most of DEGs involved in cellular transport, lignin biosynthesis and flavonoid metabolism were upregulated by N deficiency in *O*. *sativa* leaves (Fig 6). Results of real-time quantitative PCR (RT-qPCR) further verified the RNA-Seq data. Our RNA-Seq data provided a global view of transcriptomic profile of principal processes implicated in the adaptation of N deficiency in *O*. *sativa*. Furthermore, due to the status of carbohydrates, nitrogen, phosphorus and lignin contents in rice leaf under N limitation, we proposed that the function of some key genes, such as *sugar transport protein MST3-like*, *NRT2*.*1*, *laccase* and *PHO1* could be characterized in future for their potential in improving rice adaptation to low N.

## Conflicts of interest

The authors declare no conflict of interest. The founding sponsors had no role in the design of the study; in the collection, analyses, or interpretation of data; in the writing of the manuscript, and in the decision to publish the results.

## Supporting information

S1 Table Primer pairs used for RT-qPCR analysis. (XLSX)

S2 Table Summary of RNA-Seq data of *O*. *sativa* leaves under different N levels. (XLSX)

S3 Table List of DEGs in *Oryza sativa* leaves under N deficiency. (XLSX)

S4 Table GO enrichment of DEGs in response to N deficiency in *O*. *sativa* leaves. (XLSX)

S5 Table Top ten N-deficiency responsive DEGs in each pathway. (XLSX)
